# *Loranthus micranthus* Linn.: Biological Activities and Phytochemistry

**DOI:** 10.1155/2013/273712

**Published:** 2013-09-10

**Authors:** Soheil Zorofchian Moghadamtousi, Maryam Hajrezaei, Habsah Abdul Kadir, Keivan Zandi

**Affiliations:** ^1^Biomolecular Research Group, Biochemistry Program, Institute of Biological Sciences, Faculty of Science, University of Malaya, 50603 Kuala Lumpur, Malaysia; ^2^Department of Molecular Medicine, Faculty of Medicine, University of Malaya, 50603 Kuala Lumpur, Malaysia; ^3^Department of Medical Microbiology, Tropical Infectious Disease Research and Education Center (TIDREC), Faculty of Medicine, University of Malaya, 50603 Kuala Lumpur, Malaysia

## Abstract

*Loranthus micranthus* Linn. is a medicinal plant from the Loranthaceae family commonly known as an eastern Nigeria species of the African mistletoe and is widely used in folkloric medicine to cure various ailments and diseases. It is semiparasitic plant because of growing on various host trees and shrubs and absorbing mineral nutrition and water from respective host. Hence, the phytochemicals and biological activities of *L. micranthus* demonstrated strong host and harvesting period dependency. The leaves have been proved to possess immunomodulatory, antidiabetic, antimicrobial, antihypertensive, antioxidant, antidiarrhoeal, and hypolipidemic activities. This review summarizes the information and findings concerning the current knowledge on the biological activities, pharmacological properties, toxicity, and chemical constituents of *Loranthus micranthus*.

## 1. Introduction

*Loranthus micranthus *(*L. micranthus*) as member of the Loranthaceae family is the eastern Nigeria species of the African mistletoe. Mistletoes are the semiparasitic plants because they normally grow on various host trees and shrubs and they are dependent on their respective host for mineral nutrition and water, although they produce their own carbohydrates through photosynthesis [1]. Mistletoe was described as “an all purpose herb” due to its rich traditional uses and it has been widely used in ethnomedicine for various purposes, including antihypertensive, anticancer, antispasmodic, and antidiabetic, and for treatment of epilepsy, headache, infertility, menopausal syndrome and rheumatism [2, 3]. Previous studies demonstrated that composition and hence biological activities of mistletoe are dependent on harvesting period and host tree species [4–7]. Nigeria has wide distribution of mistletoes with different local names that depend on the area where they occur. *Loranthus micranthus* represents Eastern Nigeria mistletoes that mostly grow in the southeastern region of the country. It grows on various host trees including *Persia americana, Baphia nitida, Kola acuminata, Pentaclethra macrophylla, *and *Azadirachta indica *[8, 9].* L. micranthus *has been widely used in folk medicine as antimicrobial, antihypertensive, anticancer, and antidiabetic agent, for treatment of headache, infertility, epilepsy, cardiovascular diseases, menopausal syndrome, agglutination, and rheumatism, and also in conditions that generally require immunomodulatory. Some of these ethnomedicinal uses have already been supported and acclaimed by several investigations [1, 2, 10, 11]. In Nigeria and South Africa, *L. micranthus* has been widely used as ethnomedicine for treatment of hypertension, diabetes, and schizophrenia and as an immune system booster [10].

## 2. Phytochemistry

**Figure 1:**
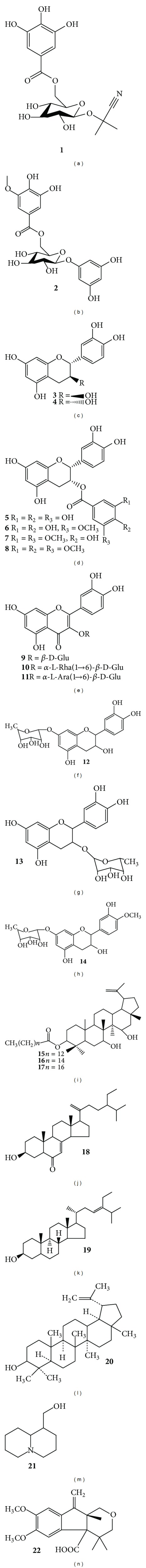
Chemical structure of chemical compounds isolated from *L. micranthus. *

Extensive phytochemical evaluations of *L. micranthus* extracts demonstrated the presence of various phytoconstituents and compounds. Crude methanolic extract from leaves of *L. micranthus* harvested from *P. americana *was found to possess terpenoids, steroids, oils, proteins, resins, flavonoids, tannins, saponins, alkaloids, reducing sugar, acidic compounds, glycosides, and carbohydrates. Alkaloids showed the highest presence in abundance. The weakly acidic fraction analysis isolated from aqueous methanolic extract of leaves of *L. micranthus* showed the presence of terpenoids, steroids, acidic compounds, flavonoids, and carbohydrate. Lower rate of flavonoids and carbohydrates elicited in comparison with methanolic extract, while the amount of terpenoids, acidic compounds, and steroids remained unchanged [28]. Chemical composition of *L. micranthus* was found to be seasonal dependent. During April, the onset of rainy season, methanolic extract of the leaves harvested from *P. Americana *showed higher concentrations of carbohydrates, acidic compounds, steroids, and alkaloids compared to July, the time for peak of rainy season. Interestingly, Tannins, saponins, glycosides, and reducing sugars were not found in July samples. However, higher amounts of flavonoids and oils were detected in July samples compared to April ones [29]. Iwalokun and colleagues [30] have investigated the phytochemicals such as *n*-butanol, chloroform, ethyl acetate, and water fractions of the methanolic extract of *L. micranthus* leaves of Kola nut tree (*K. acuminata*). Moderate and high levels of steroids and terpenoids were detected in *n*-butanol fraction, while phenolics, reducing sugars, and tannins were detected in all fractions together with moderate level for phenolics and tannins in chloroform fraction. Indeed, flavonoids and saponins were only present in ethyl acetate and water fractions, respectively. They have demonstrated that terpenoids were present in low-to-moderate levels in chloroform and water fractions, while they were not detected in ethyl acetate fraction. Phytochemical studies on *L. micranthus* leaves harvested from six different host trees, namely, *P. americana, B. nitida, K. acuminata, P. macrophylla, A. indica,* and *Irvingia gabonensis,* revealed that alkaloids are preponderant in the extracts of *K. acuminata, P. Americana,* and *I. gabonensis*. Moreover, the phytochemical constituent host dependency was also shown [31, 32]. A study on petroleum ether extract of *L. micranthus* leaves parasitic on *P. americana* harvested at different seasons (January, April, July, and November) showed only presence of alkaloids in April and July and proved harvesting period dependency in phytochemicals of *L. micranthus *[33]. In several studies attempting to identify the active compounds responsible for various biological activities of *L. micranthus*, especially immunomodulatory activity, a variety of compounds (Table 1) were isolated and their structures were characterized (Figure 1).

## 3. Biological Activities

Fractions, pure compounds, and crude extracts of plants are precious and crucial resources for effective molecules in treatment of different diseases and ailments in animal and human [34]. *L. micranthus *has been found to possess antidiabetic, antimicrobial, antihypertensive, hypolipidemic, antioxidant, antidiarrhoeal, and immunomodulatory activities. A study by Edem and Usoh [35] demonstrated that the use of *L. micranthus* is safe and without adverse biochemical effects or hepatocellular damages in rats. They have administered different doses of the water extract of *L. micranthus *leaves from 275 to 827 mg/kg for 21 days on male albino Wistar rats. Blood samples analysis did not reveal any significant changes in the level of protein, urea, glucose, bilirubin, cholesterol, alkaline phosphatase, and aspartate transaminase. However, significant reduction was observed in the level of alanine transaminase enzyme by using 551 and 827 mg/kg of the extract. However, an *in vitro* study on aqueous leaf extract of *L. micranthus* indicated cytotoxic, genotoxic, and mitodepressive effects of that extract against *Allium cepa *root cells especially at dose beyond pharmacological range [36]. They showed a significant inhibition of root growth with an effective concentration (EC_50_) of 28.2 mg/mL. The extract revealed dose-dependent decrease in mitotic index by 2.4–27.4% using a range from 5 to 40 mg/mL of the extract except at 2.5 mg/mL in which 11.9% elevation was reported compared to 50% decrease for sodium azide used at 100 *μ*g/mL as positive control. Hence, for safety reasons, using the lower concentrations of *L. micranthus *for human phytomedicine in prolonged use coupled with *in vivo *genotoxic tests is suggested [36].

### 3.1. Immunomodulatory  Activity

Stimulation of human immune system has been identified as a possible pathway to inhibit the progression of some diseases without eliciting adverse side effects [37, 38]. However, immune system stimulation is a desired response if overall process culminates with cure or faster convalescence from sickness [39, 40]. One folkloric use of *L. micranthus *is due to its ability to enhance the immune system with concomitant quicker convalescence [10]. An *in vivo* study on aqueous-methanol extract from *L. micranthus* leaves harvested from five different host trees has investigated the immunomodulatory activity in rat and mice models, including the cyclophosphamide-induced myelosuppression (CIM) test, the humoral mediated antibody titration (AT) test, total and differential leukocyte count (TLC and DLC), and the cellular mediated delayed-type hypersensitivity reaction (DTHR) test [10]. According to the results, the extracts (with overall order of activity from *K. acuminate *> *Citrus *spp. > *P. Americana *> *Parkia biglobosa *> *P. macrophylla*) were found to be potent and safe complementary or alternative medicines to cure the immunodeficiency conditions without any toxicity (LD_50_ values > 5000 mg/kg for acute toxicity tests). Leaves extract parasitic on *K. acuminate *at a dose of 200 mg/kg caused 139.69% stimulation of total leukocyte in mice compared to 75.35% increase for levamisole (25 mg/kg) as a standard immunostimulatory drug. At dose of 100 mg/kg, it also showed 175% stimulation on DTHR in immunocompetent mice compared to 122.50% increase for levamisole as positive control. 71.35% and 81.53% of primary and secondary stimulation on antibody titration in rats at a dose of 100 mg/kg of leaves extract also exhibited the significant immunomodulatory effect compared to 24.50% and 0.40% of primary and secondary stimulation for levamisole (25 mg/kg), respectively [10, 41]. Results from another study by the same group showed that ethanol and *n*-hexane leaves extracts parasitic on *P. americana* at doses from 100 to 400 mg/kg also exhibited a dose-dependent immunomodulatory activity assessed by DTHR and CIM models in mice. Results showed over 170% of stimulation for both extracts at 400 mg/kg dose [42]. The immunomodulatory analysis of five different extracts of *L. micranthus* leaves, parasitic on *K. acuminata*, namely, *n*-hexane, chloroform, ethyl acetate, acetone, and methanol by DTHR test in experimental mice and their phytochemical evaluation of the fractions, demonstrated that the most active fractions were either mainly terpenoidal, flavonoidal, or steroidal [43]. The results also confirmed the most significant immunostimulatory effects for chloroform extract with 311.11% and 122.22% enhancement in stimulation using 250 and 500 mg/kg of the extract, respectively. The compounds **12**–**14** at dose of 100 *μ*g/mL showed increasing effects on C57BL/6 mice splenocytes proliferation with 91.49%, 95.17%, and 94.23% stimulation values, respectively, compared to 16.09% stimulation for 10 *μ*g/mL of lipopolysaccharide (LPS) as positive control. However, compounds **12**, **13** and **14** exhibited moderate stimulatory effect on CD69 molecule expression [22]. The compound **15** with dose of 100 *μ*g/mL exhibited a weak immunostimulatory activity by inducing 24.44% and 86.98% stimulation of mice splenocytes proliferation and early activation of CD69 molecule, respectively. In addition, it also showed a nonsignificant effect on IL-8 receptor expression [44]. The steroids compounds of **18** and **19** at a dose of 100 *μ*g/mL exhibited significant immunostimulatory activity on the C57BL/6 mice splenocytes with 46% and 43% stimulation, respectively, compared to 7.69% increase for the negative control, although CD69 expression assay revealed minimal stimulation. The compounds **16** and **17** with the same concentration showed weaker stimulations of 30% and 29%, respectively, on the C57BL/6 mice splenocytes [24]. Also, a mild immunostimulatory activity was observed for compound **20** when it was tested on C57BL/6 splenocytes [25]. However, 69.84% and 56.34% stimulation elicited from compounds **21** and **22** at a dose of 25 *μ*g/mL for proliferation analysis on mice splenocytes (C57BL/6) compared to 7.58% value for unstimulated control. The CD69 expression assay also exhibited significant proliferation values of 2.71% and 2.31% for compounds of **21** and **22**, respectively [27].

### 3.2. Antidiabetic Activity

Diabetes mellitus is one of the prevalent and serious chronic diseases around the world [45]. Therefore, finding the promising ways to improve the quality of life for diabetic patients and preventing or reversing diabetic complications necessitate investigations among the arsenal of herbs [46]. Osadebe and colleagues [32] have studied the anti-diabetic activity of the methanolic extracts of leaves of *L. micranthus *parasitic on *P. americana, B. nitida, K. acuminata, P. macrophylla, *and *A. indica*. The extracts were found to possess significant dose-dependent antihyperglycemic effects in alloxan-induced diabetic albino and normoglycemic rats, respectively. The maximum activity of the methanolic extract of *L. micranthus* (400 mg/kg) harvested from *P. americana *on alloxan-induced diabetic rats showed 82.59% reduction of blood sugar level at 24 h after administration determined by *o*-toluidine spectrophotometric method which is statistically comparable with the 83.34% of reduction for glibenclamide as a positive control. Methanolic extracts of *L. micranthus *from five different host trees did not show any toxicity according the acute toxicity tests in mice (LD_50_ values of 11650, 11650, 5900, 5900, and 5900 mg/kg for *P. americana, B. nitida, K. acuminata, P. macrophylla, *and *A. indica, *resp.). The leaves of *L. micranthus *parasitic on *K. acuminata*, *A. indica, *and *B. nitida *showed more significant antihyperglycemic activity among the other host trees investigated. The results demonstrated that the antidiabetic effect of the extract was found to be dependent on the host plant species. The weakly acidic fraction of the aqueous methanol extract of the leaves of *L. micranthus* parasitic on *P. americana *revealed anti-diabetic activity in alloxan-induced diabetic rats. The low-acidic fraction at the dose of 400 mg/kg reduced 66.60% of blood sugar level of alloxan-induced diabetic rats at 24 hours after administration [28]. However, Osadebe and colleagues [29] have studied the seasonal variation for the anti-diabetic effect of the aqueous methanolic extract of the leaves of *L. micranthus, *parasitic on *P. americana,* in alloxan-induced diabetic rats. The study demonstrated that anti-diabetic effect of the extract is seasonal and dose dependent with the highest activity being at the peak of the rainy season. The results revealed 39.2% and 47.5% fasting blood sugar reduction after 6 hours consumption of 400 mg/kg of April and July samples, respectively, with 8.3% difference due to effect of seasonal variation on chemical content of leaves. Higher concentration for flavonoids in peak of the rainy season compared to the onset of rainy season could be responsible for the observed higher anti-diabetic activity in July. However, there is no data available for the particular bioactive compound(s) from *L. micranthus* with known mechanism for anti-diabetic activity. Therefore, this is an open area for the future research around this plant. Uzochukwu and Osadeb [47] have studied a comparative evaluation of antidiabetic activities of crude methanolic extract and flavonoids extract of *L. micranthus *harvested from *K. acuminata* in alloxan-induced diabetic rats. Results revealed that flavonoids extract (200 mg/kg) showed significant anti-diabetic effect within one hour of administration, while the methanolic extract (200 mg/kg) showed the significant antidiabetic effect within three hours of administration. In addition, phytoconstituents of other members of Loranthaceae plants possessing antihypertensive activity are interestingly similar to *L. micranthus *[48–50].

### 3.3. Antimicrobial Activity

Using medicinal plants as antimicrobial remedy has a long history in both developed and developing countries. In addition, in contemporary medicine because of unreasonable and indiscriminate consumption of antimicrobial drugs, the infectious microorganisms have developed resistance. Therefore, controlling the existing infectious diseases necessitates new alternative antimicrobial drug regimens [51]. Osodebe and Ukwueze have presented the wide range of data regarding the antimicrobial activities of* L. micranthus *[31]. A study on different extracts of *L. micranthus* leaves harvested from *K. acuminata *such as methanolic, ethanolic, chloroform, and petroleum ether extracts demonstrated the antibacterial activities for all tested extracts against *Bacillus subtilis, Escherichia coli,* and *Klebsiella pneumonia,* while only petroleum ether extract exerted antifungal activity against *Aspergillus niger* and *Candida albicans* with the MIC of 4.30 and 1.73 mg/mL, respectively, and no activity against *Klebsiella pneumonia*. The methanolic extract exhibited the most potent antibacterial effect against *Bacillus subtilis* and *Escherichia coli* with MIC of 1.58 and 1.48 mg/mL, respectively, compared to the other tested extracts [52]. The ethyl acetate fraction of crude petroleum ether extract of *L. micranthus* leaves parasitic on *K. acuminata* also showed inhibitory activities against *Candida albicans* and *Bacillus subtilis* [53]. A comparative investigation on antimicrobial activities of *L. micranthus* leaves and its phytochemicals from six different host trees, including *P. americana, B. nitida, K. acuminata, P. macrophylla, A. indica,* and *I. gabonensis, *indicated the relative significant antibacterial activities for *L. micranthus *parasitic on *K. acuminata* and *P. americana*. Alkaloids in these three species were found to be most abundant. Preponderance of alkaloids in these species could be responsible for the marked antimicrobial activities. The leaves extract parasitic on *P. Americana *exerted more potent antibacterial activity against *Pseudomonas aeruginosa *compared to amoxicillin, while the extracts from *A. indica, P. macrophylla, *and *I. gabonensis* exhibited stronger antibacterial activity against *Staphylococcus aureus* in comparison with amoxicillin [31]. Effect of different harvesting seasons (January, April, July, and November) on antimicrobial activity of petroleum ether extracts from *L. micranthus* leaves, parasitic on *P. americana,* and its phytochemicals, also demonstrated seasonal dependency. Alkaloids as one of the major groups of antibacterial candidates was only found in July and April. Antibacterial activity against *Salmonella typhi* and *Bacillus subtilis* was markedly lower in January samples compared to the extracts from the other months' samples [33, 54]. In addition, no significant antifungal activity for methanolic extracts of *L. micranthus* leaves harvested from *K. acuminata*, *P. Americana,* and *I. gabonensis *was demonstrated against *A. niger *(MIC > 3 mg/mL) and *C. albicans *(MIC > 4 mg/mL) compared to ketoconazole (MIC < 1 mg/mL) as an approved antifungal agent [55]. To sum up, it has been proven that leaf extracts of *L. micranthus* elicited significant antibacterial activity against *B. subtilis, P. aeruginosa, E. coli, *and *Staph. aureus* without significant antifungal activity [56]. 

### 3.4. Antihypertensive Activity

Deaths because of hypertension arise out of cardiovascular and cerebrovascular complications including cardiac arrest, stroke, myocardial infarction, congestive heart failure, and end-stage renal disease [57]. Early detection and commencement of treatment are vital for prevention and delaying the aftermaths and enhance the chance of longer life for afflicted patients [58]. In the last few decades, plants have still remained as a rich source for efficacious, safe, and cost-effective antihypertensive drugs [59]. *L. micranthus* is one of the plants identified with antihypertensive activity for humans and animals in sub-Saharan Africa [60]. Aqueous extract of *L. micranthus* (1.32 g/kg per day) exhibited hypotensive effect on normotensive and spontaneous hypertensive rats [61]. A noteworthy reduction in the mean arterial pressure (MAP) was obtained in both groups of rats without effect on the urinary flow rate or the respiratory rate. In addition, the level of total cholesterol exhibited significant reduction on days 6, 7, and 8 [61]. Indeed, methanolic extract of leaves of *L. micranthus *demonstrated a dose-dependent inhibition of blood pressure increase in adrenaline-induced hypertensive rat. Iwalokun and colleagues [30] studied the activity of *n*-butanol, chloroform, ethyl acetate, and water fraction of the methanolic extract of *L. micranthus* leaf harvested from *K. acuminata *on pressor-induced contraction of rat aorta smooth muscles. *N*-Butanol fraction showed the highest dose-dependent inhibitory activity (EC_50_ = 0.65 mg/mL and smooth muscle relaxation of 75.2%) on rat aorta precontracted with norepinephrine and KCl, followed by decreasing order by water, chloroform, and ethyl acetate fractions. The same order of activity was observed in the ability of these fractions to reduce cardiac arginase with 11.7% reduction for *n*-butanol fraction and to raise serum nitric oxide with 55% elevation for *n*-butanol fraction in mice orally administrated 250 mg/kg of fractions for 21 days. Arginase was found to be a diagnostic indicator for cardiovascular diseases including hypertension [62]. Enhanced activity of nitric oxide is a critical factor to reduce the vascular resistance and blood pressure that increased in hypertensive rats and humans [63, 64]. Cardiac arginase reduction, vasorelaxation, antiatherogenic events, and nitric oxide (NO) elevation were found to be responsible for antihypertensive activity of *L. micranthus.* Moderate and high abundance of steroids and terpenoids in *n*-butanol fraction strongly suggested that these phytoconstituents could be responsible for observed vasorelaxant and antiatherogenic activity of *n*-butanol fraction. Iwalokun and colleagues [30] have reviewed the possible mechanisms of antihypertensive activity of *L. micranthus.*

### 3.5. Antioxidant Activity

The role of free radicals in many diseases has been proven by recent developments in biomedical sciences. Cellular damage caused by free radicals is possibly responsible for many degenerative diseases including heart disease, cancer, brain dysfunction, and immune system decline [65]. Phenolic compounds are potent scavengers of free radicals [66]. The antioxidant activity of compounds **1**–**11** isolated from methanol extracts of *L. micranthus* leafy twigs was investigated by 2,2-diphenyl-1-picrylhydrazyl (DPPH) assay. All the tested compounds revealed significant antioxidant effect (IC_50_ = 23.8–50.1 *μ*M) compared to chlorogenic acid as a positive control (IC_50_ = 67.9 *μ*M). Compounds **10** and **11** exhibited the most significant antioxidant activity [12]. The results of DPPH assay on compounds **12**–**14** revealed significant antioxidant potentials with EC_50_ of 55.42, 58.45, and 59.71 mg/mL, respectively, compared to ascorbic acid as a positive control with EC_50_ = 17.6 mg/mL [22].

### 3.6. Antidiarrhoeal Activity

Diarrhoea due to the risk of sever potentially fatal dehydration can be a serious complication in infants and elderly people [67]. About 2.2 million people, mostly infants and children below 5 years, are victims of diarrhea annually [68]. Because of the adverse effects of some existing antimotility medicines after prolonged use, many studies have been done for an alternative remedy among traditional medicinal herbs [69, 70]. *In vivo *study on methanolic extract of *L. micranthus *leaf harvested from six different host trees, including *P. americana, B. nitida, K. acuminata, P. macrophylla, C. sinensis, *and *I. gabonensis, *indicated antimotility effect in rats with castor-oil-induced diarrhoea. Methanolic extract of *L. micranthus *parasitic on *P. macrophylla* (200 mg/kg) showed the most significant decrease on defecation (93.33%) 4 hours after administration compared to atropine sulphate (2 mg/kg) as a positive control (80%). It also significantly inhibited gastrointestinal transit by 67.6% which is more than atropine sulphate (26.4%) [9].

### 3.7. Hypolipidemic Activity

The serum lipid analysis in mice orally was administered with 250 mg/kg of *n*-butanol, chloroform, ethyl acetate, and water fractions of the methanolic extract of *L. micranthus *harvested from *K. acuminata *for 21 days indicated with decrease in cholesterol and triglyceride with the highest activity for *n*-butanol fraction. Results demonstrated lower total cholesterol, triglycerides (TAG), and LDL-cholesterol levels particularly for *n*-butanol and water fractions after 21 days without significant changes for HDL-cholesterol. On day 21, *n*-butanol fraction significantly reduced TAG level (138.5 ± 3.4 versus 152.8 ± 0.6 mg/dL). Total cholesterol and LDL-cholesterol levels also reduced from 114.3 ± 3.5 mg/dL to 91.9 ± 0.4 mg/dL and from 42.2 ± 4.3 mg/dL to 23.7 ± 0.9 mg/dL by *n*-butanol fraction on the day 21, respectively [30].

## 4. Conclusion

The overview of scientific investigations on *L. micranthus *showed various biological activities for this plant. The phytochemical constituents and the activity of the medicinal values of *L. micranthus *are strongly dependent on harvesting season and host species trees. Phytochemicals and compounds isolated from *L. micranthus* are in interest for the further investigations towards application of this plant as anti-diabetic, antibacterial, antihypertensive, hypolipidemic, antioxidant, antidiarrhoeal, and immunomodulatory medicines. Further *in vivo *genotoxic tests of this plant can be also beneficial for the safety approval for therapeutic applications.

## Figures and Tables

**Table 1:** Phytochemistry and bioactivity of compounds isolated from *L. micranthus. *

Plant part	Compound	Code	Chemical Category	Biological activity	Host tree	Reference
Leafy twigs	Linamarin gallate	1	Phenolics glycoside	Antioxidant activity	*Hevea brasiliensis *	[12]
	Walsuraside B	2	Phenolics glycoside	Antioxidant activity	*Hevea brasiliensis *	[12]
	Catechin	3	Polyphenol	Antioxidant activity	*Hevea brasiliensis *	[13]
	Epicatechin	4	Polyphenol	Antioxidant activity	*Hevea brasiliensis *	[14]
	Epicatechin 3-O-gallate	5	Polyphenol	Antioxidant activity	*Hevea brasiliensis *	[15]
	Epicatechin 3-O-(3-O-methyl) gallate	6	Polyphenol	Antioxidant activity	*Hevea brasiliensis *	[16]
	Epicatechin 3-O-(3,5-O-dimethyl) gallate	7	Polyphenol	Antioxidant activity	*Hevea brasiliensis *	[17]
	Epicatechin 3-O-(3,4,5-O-trimethyl) gallate	8	Polyphenol	Antioxidant activity	*Hevea brasiliensis *	[18]
	Quercetin 3-O-**β**-D-glucopyranoside	9	Polyphenol	Antioxidant activity	*Hevea brasiliensis *	[19]
	Rutin	10	Polyphenol	Antioxidant activity	*Hevea brasiliensis *	[20]
	Peltatoside	11	Polyphenol	Antioxidant activity	*Hevea brasiliensis *	[21]

Leaves	-(-) catechin-7-O-rhamnoside	12	Polyphenol	Immunomodulatory and antioxidant activity	*Kola acuminata *	[22]
	-(-) catechin-3-O-rhamnoside	13	Polyphenol	Immunomodulatory and antioxidant activity	*Kola acuminata *	[22]
	4′-methoxy-catechin-7-O-rhamnoside	14	Polyphenol	Immunomodulatory and antioxidant activity	*Kola acuminata *	[22]
	7**β**, 15**α**-dihydroxy-lup-20(29)-en-3**β**-O-palmitate	15	Triterpenoid ester	Immunomodulatory activity	*Kola acuminata *	[23]
	7**β**, 15**α**-dihydroxy-lup-20(29)-en-3**β**-O-stearate	16	Triterpenoid ester	Immunomodulatory activity	*Kola acuminata *	[23]
	7**β**, 15**α**-dihydroxy-lup-20(29)-en-3**β**-O-eicosanoate	17	Triterpenoid ester	Immunomodulatory activity	*Kola acuminata *	[23]
	stigmast-7,20(21)-diene-3**β**-hydroxy-6-one	18	Steroid	Immunomodulatory activity	*Kola acuminata *	[24]
	3**β**-hydroxystigmast-23-ene (stigmast-23-en-3**β**-ol)	19	Steroid	Immunomodulatory activity	*Kola acuminata *	[24]
	Lupeol	20	Triterpenoid	Immunomodulatory activity	*Kola acuminata *	[25]
	Lupinine	21	Alkaloid	Immunomodulatory activity	*Kola acuminata *	[26, 27]
	Loranthoic acid	22	Steroid	Immunomodulatory activity	*Kola acuminata *	[26, 27]
